# Improving access to public physical activity events for disadvantaged communities in Australia

**DOI:** 10.1186/s12889-022-13981-5

**Published:** 2022-08-13

**Authors:** Janette L. Smith, Lindsey J. Reece, Catriona L. Rose, Katherine B. Owen

**Affiliations:** 1 Prevention Research Collaboration, School of Public Health, Faculty of Medicine and Health, The University of Sydney, Level 6, Charles Perkins Centre, Camperdown, NSW 2006 Australia; 2 NSW Biostatistics Training Program, NSW Ministry of Health, St Leonards, NSW 2065 Australia

**Keywords:** Physical activity, Health inequalities, Socioeconomic disparities, Geospatial analysis, Parkrun, Health promotion

## Abstract

**Background:**

Physical activity has numerous health benefits, but participation is lower in disadvantaged communities. ‘parkrun’ overcomes one of the main barriers for disadvantaged communities, the cost of activities, by providing a free, regular community-based physical activity event for walkers, runners and volunteers. This study assesses equity of access (in terms of distance to the nearest parkrun) stratified by socioeconomic deprivation, and identifies the optimal location for 100 new events to increase equity of access.

**Methods:**

We combined information about population location and socioeconomic deprivation, with information about the location of 403 existing parkrun events, to assess the current level of access by deprivation quintile. We then used a two-step location-allocation analysis (minimising the sum of deprivation-weighted distances) to identify optimal regions, then optimal towns within those regions, as the ideal locations for 100 new parkrun events.

**Results:**

Currently, 63.1% of the Australian population lives within 5 km of an event, and the average distance to an event is 14.5 km. A socioeconomic gradient exists, with the most deprived communities having the largest average distance to an event (27.0 km), and the least deprived communities having the best access (living an average 6.6 km from an event). Access improves considerably after the introduction of new event locations with around 68% of the population residing within 5 km of an event, and the average distance to the nearest event approximately 8 km. Most importantly, the improvement in access will be greatest for the most deprived communities (now an average 11 km from an event).

**Conclusions:**

There is a socioeconomic gradient in access to parkrun events. Strategic selection of new parkrun locations will improve equity of access to community physical activity events, and could contribute to enabling greater participation in physical activity by disadvantaged communities.

**Supplementary Information:**

The online version contains supplementary material available at 10.1186/s12889-022-13981-5.

## Background

Insufficient physical activity is a modifiable risk factor for poor health outcomes [1], accounting for 2.5% of the total disease burden in Australia [2]. Globally, 27.5% of adults and 81.0% of adolescents (aged 11–17 years) do not meet minimum physical activity guidelines [3, 4]. The WHO’s Global Action Plan on Physical Activity (GAPPA) [5] highlighted the need to “implement regular mass participation initiatives in public spaces, engaging entire communities, to provide free access to enjoyable and affordable, socially- and culturally-appropriate experiences of physical activity” (p29). GAPPA [5] also highlights the importance of equity across the life course, requiring countries to prioritise addressing disparities and reducing inequalities in their implementation of the action plan to achieve the goal of a 15% reduction in physical inactivity by 2030. Further, in 2021, the World Health Organisation [6] published an advocacy brief calling for stronger multisectoral action to address inequities in access and opportunities for physical activity. There is a need for scaled-up, effective and equitable interventions that will increase physical activity across the population.

‘parkrun’ is one potential solution. parkrun is a free, regular community-based physical activity event for walkers, runners and volunteers, beginning in London in 2004 and now involving more than 350,000 people each week in 22 countries across the world (see parkrun.com). parkrun is an attractive way to engage communities in more physical activity, as it removes or reduces many of the barriers to engaging in physical activity, including the high cost of engaging in some forms of exercise, a lack of a suitable place to exercise, and poor social support to exercise [7–9]. However, current parkrun published data shows that while parkrun has good reach overall, levels of engagement tend to be lower in those living in disadvantaged areas [10, 11].

A recent paper from Schneider et al. [12] examined access to English parkrun events and found that, contrary to expectations, access to parkrun events was best for areas with greater socioeconomic deprivation. They also identified 200 public green spaces which would considerably improve access to parkrun across England. In this study, we perform similar analyses for Australia. As of July 2021, there were 403 public 5 km events in Australia, mostly located in densely populated coastal areas and cities, with poor access (that is, a long distance to travel to the nearest event) for communities residing elsewhere in Australia. We are the first to assess the current levels of access to Australian parkrun events by socioeconomic deprivation quintiles; we also use geospatial analysis to identify the optimal locations for 100 new parkrun events, with the aim of reducing distances to the nearest event, particularly for those in the most disadvantaged communities in Australia.

## Methods

### Data sources

#### Population location

All analyses were conducted at the level of Statistical Area Level 1 (“SA1”, 2016 definition). These are geographical areas defined by the Australian Statistical Geography Standard of the Australian Bureau of Statistics [13] and are the smallest unit at which census data is released publicly. Australia is divided into 57,523 such units with no gaps or overlaps; however, 33 of these are non-spatial special purpose codes, so our analysis used the remaining 57,490 SA1 areas. Each SA1 has a population of approximately 450 people on average (range: 0–10,048) but the area covered (in square km) has a large range: on average, each SA1 covers 133.7 square km, with a range from 0.002 (in inner Sydney) to 328,261 square km (in the Western Australia outback). We retrieved spatial information about SA1 areas [13], the predicted population for 2020 (the most recent year available) living within each SA1 [14], and the Index of Relative Socio-economic Disadvantage (IRSD, see below) for each SA1. We also obtained spatial information about Statistical Area Level 2 (“SA2”), which are comprised of whole SA1 areas and represent communities which interact socially and economically [13].

#### Relative socioeconomic disadvantage

Area-level socioeconomic status was obtained for all SA1 areas and categorised using the Socio-Economic Index for Area (SEIFA), specifically the Index of Relative Socio-Economic Disadvantage, or ‘IRSD’ [15]. This index calculates a relative disadvantage score for each SA1, then determines the percentile ranking for each SA1. In our analyses a score of 100 reflected the most disadvantaged areas, while a score of 1 reflected the least disadvantaged areas. Because the IRSD was not available for 2,495 SA1 areas, and complete data was required for all SA1s for the location-allocation algorithm, we used, in order of preference, the IRSD for the SA2 area, the postcode, or the median (50).

#### Location of current events

parkrun Australia supplied the latitude and longitude coordinates of all 403 public 5 km parkrun events currently in operation or planned to start by July 2021.

### Procedures

The two main variables of interest in analyses were access to parkrun and relative socioeconomic disadvantage (IRSD) for each SA1 area. Access was defined by the geodesic distance (i.e., distance “as the crow flies”) between the centroid (the geographical centre) of each SA1 to the location of the nearest event. We calculated the distance from each of the 57,490 SA1 centroids to each of the 403 current parkrun locations, and selected the shortest distance to determine the name of, and distance to, the nearest event for each SA1. We summarised the current level of access to parkrun events in terms of the mean population and number of SA1s in the catchment area for each event (i.e., for how many SA1s, and how many people, is a given event the nearest event?). We also stratified distance to the nearest event by IRSD quintiles.

For locations to potentially place new parkrun events, we developed a two-step process to first select a general region (at the SA2 level) to place a new event, then to select suitable towns in the region via two different methods. In more detail: in the first step, we used location-allocation analysis, a method of choosing which among several alternative new locations for a resource will most effectively supply demand points. We used the deprivation-weighted distance minimising method to identify which 100 of the 2292 SA2 areas to place new events to provide best access to the greatest number of SA1 areas, weighted by socioeconomic disadvantage. Specifically, and following Schneider et al. [12], for each candidate SA2 centroid, we calculated how a new parkrun event at that location would alter the sum of distances, weighted by the square of IRSD, from all SA1 centroids to that candidate SA2, and selected the candidate that would minimise this sum. This location was then added to the list of existing events, and the process repeated until 100 new regions were identified.

However, examination of the results of this analysis revealed that the centroids of the selected SA2s were often not in a suitable location to hold an event; in some very large SA2s in central Australia, the centroid may be tens or hundreds of km from the nearest town, and lack both the infrastructure (e.g., large parks, playing fields) and critical mass of population required to start and sustain a new event. Therefore, a second step was necessary to identify possible locations at the level of towns (rather than SA2 regions). In the second step, we considered each SA1 within the 100 selected SA2 regions, and used two methods to select individual SA1s within that SA2 which may be suitable for an event. Method 1 used deprivation-weighted distance as well as population density in that it (1) considered only those SA1s with population density more than 10 people per square km (an arbitrary cut-off that was sufficient to identify townships in our analyses), (2) calculated the distance from those SA1 centroids to the centroid of the SA2 area of which they are part, (3) multiplied this distance by the square of the IRSD of that SA1 (on its original scale, such that the most disadvantaged communities had a percentile and weight of 1, and the least disadvantaged had the highest rank and multiplier), and (4) selected the SA1 with the minimum weighted distance. Effectively, this method replicates the location-allocation algorithm used in the first step, and favours a SA1 close to the SA2 centroid; if two SA1s were equally distant to the SA2 centroid, then the more disadvantaged SA1 would be selected.

Method 2 simply identified the most densely populated SA1 within the SA2, regardless of its distance from the SA2 centroid or the disadvantage of the people living within that area; in many but not all cases, this selected an SA1 in the same town as the first method. We consider the analysis with Method 1 (deprivation-weighted distance) as the major analysis, with those identified by Method 2 as supplementary locations that could be considered in case the town identified by Method 1 was unsuitable for any reason (e.g., did not have a suitable green space). We provide detailed data on improvements in access after adding new events at these locations, both overall and by IRSD quintile.

Although we use geographic information about human population as well as relative socioeconomic disadvantage, this information is publicly available and at an aggregate level at which no individual could be identified, nor consent reasonably sought from participants. The research was approved by the parkrun Research Board (202,112) and the Human Research Ethics Committee at the University of Sydney (2019/992); the views, thoughts, and opinions expressed in the manuscript belong solely to the authors, and do not necessarily reflect the position of parkrun or the parkrun Research Board. The R code used to generate the results are included as an online appendix.

## Results

### Access to current events

Approximately 4.9%, 63.1% and 85.5% of the Australian population lived within 1 km, 5 km and 10 km of an event, respectively. Only 6.2% lived more than 25 km from an event. The largest distances to an event were for people living on the Cocos (Keeling) Islands, Christmas Island, and Norfolk Island, respectively 2,314 km, 1,634 km and 1,400 km away from the nearest event on mainland Australia. The existing parkrun events are the closest event for an average of 143 SA1 catchment areas, serving on average 63,765 people (see Table 1).

**Table 1:** Descriptive statistics of SA1 areas and existing Australian parkrun events

Variable	Mean	SD	Median	IQR	Range
SA1s (*n* = 57,490)					
Population	447	285	423	321–537	0–10,048
Distance in km to nearest current event	14.5	61.2	4.1	2.5–7.3	0.04–2318.2
Parkrun events					
Catchment area population^a^	63,765	62,031	48,885	24,074–80,485	1,004–499,434
Catchment area SA1s^a^	143	124	111	63–179	4–948

^a^total population/number of SA1s for which a given parkrun event is the nearest

In the stratified analysis of access by socioeconomic disadvantage, access was best for those in the least deprived quintile, being an average 6.6 km from the nearest event, and poorest for the most deprived quintile, being an average 27.0 km from the nearest event (see Table 2).

**Table 2:** Distance in km to the nearest current Australian parkrun event, and after 100 new events are set up in the locations identified by the three methods, by the Index of Relative Socioeconomic Deprivation (IRSD) quintile

	**Current situation**					**First approximation: SA2 centroid**					**Second step: deprivation-weighted distance**					**Second step: most densely populated**				
IRSD subset	Mean	SD	Median	IQR	Range	Mean	SD	Median	IQR	Range	Mean	SD	Median	IQR	Range	Mean	SD	Median	IQR	Range
Least deprived	6.55	29.24	3.72	2.31–5.73	0.10–669.75	4.71	6.04	3.70	2.29–5.61	0.10–216.60	4.70	6.04	3.69	2.30–5.59	0.10–216.54	4.70	6.26	3.69	2.28–5.60	0.00–214.97
Less deprived	10.21	43.53	3.90	2.38–6.54	0.10–1403.76	6.66	14.91	3.82	2.35–6.10	0.10–579.70	6.62	14.90	3.80	2.35–6.04	0.10–579.70	6.61	14.83	3.81	2.34–6.04	0.00–579.70
Median deprived	13.80	60.12	4.23	2.51–8.43	0.04–2318.23	7.96	15.24	3.99	2.45–6.93	0.04–435.67	7.90	15.40	3.94	2.44–6.85	0.04–435.67	7.83	15.04	3.94	2.41–6.86	0.00–435.67
More deprived	15.05	55.87	4.37	2.56–9.17	0.05–1641.27	8.88	16.37	3.86	2.38–7.08	0.05–302.57	8.82	16.73	3.80	2.34–6.93	0.00–302.57	8.76	16.34	3.81	2.32–6.99	0.00–302.57
Most deprived	26.97	94.48	4.44	2.53–9.14	0.07–2316.56	11.37	26.55	3.50	2.13–6.64	0.00–372.13	11.03	26.34	3.38	2.03–6.19	0.00–357.72	11.30	29.80	3.39	2.06–6.19	0.00–565.74
Overall	14.54	61.17	4.09	2.45–7.28	0.04–2318.23	7.92	17.28	3.77	2.32–6.32	0.00–579.70	7.82	17.3	3.72	2.28–6.21	0.00–579.70	7.85	18.28	3.72	2.28–6.22	0.00–579.70

### Selected new sites

**Fig. 1:**
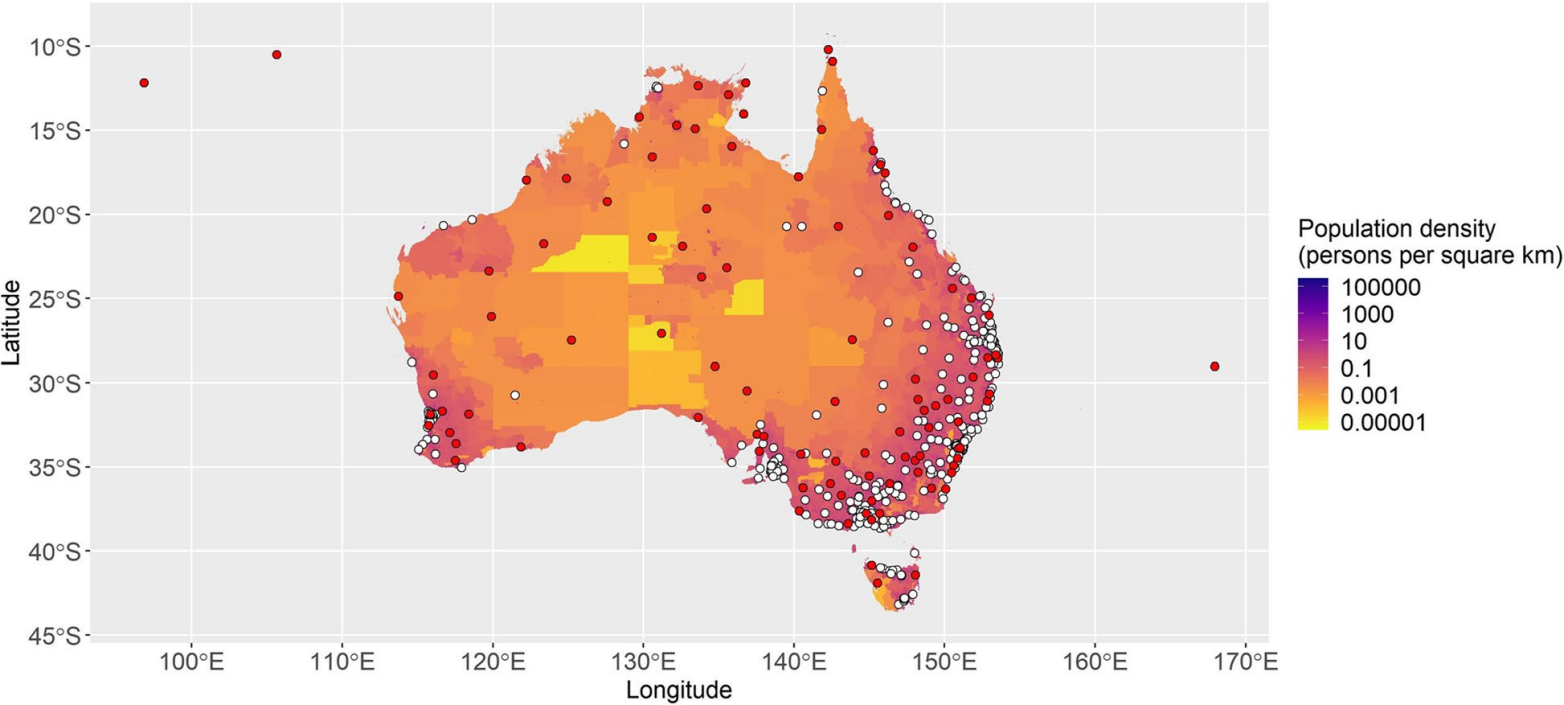
Map of Australia showing the location of 403 current parkrun events (white) and 100 proposed events (red). Note that the areas of highest population density are also the areas where most current events are located. Information about the 100 new locations, as well as more detailed maps of regional and greater capital city areas within each state, are supplied in Supplementary Material

Figure 1 maps the location of 403 current public events (white) and 100 new proposed events (red, at the SA2 centroid) across Australia. Population density is mapped for each SA1 area, where yellow represents low population density and purple the greatest population density. We also provide a high-resolution, interactive map for online viewing, which shows the location of selected towns via Method 1 and Method 2 also. For our online map resource, white circles indicate the location of current parkrun events (at July 2021); red circles indicate the centroid of the SA2s selected in the first step of the analysis, together with the name of the SA2. Yellow circles indicate the location of the SA1 closest to the SA2 centroid, weighted by IRSD, while green circles indicate the most densely populated SA1 within the selected SA2 area. Numbers indicate the order in which the SA2 was selected, and all are also labelled by the name of the SA2.

Current events are evenly divided between capital city and regional areas, with most in the most populous states (see Supplementary Table 1 for more detail). However, the location-allocation algorithm overwhelmingly selected regional sites for new events (91); new greater capital city events were selected only for Sydney (4), Perth (2) and Melbourne (3). New South Wales had the most new event locations selected (31), while the Australian Capital Territory was already well-covered and did not have any new sites selected.

### Access after new events are set up

Access would be considerably improved by setting up new events in the SA2 centroid locations; our second stage analysis provides very similar improvements in access, but with the added bonus of allowing the identification of 1–2 specific towns.

If new parkrun events are set up in the 100 locations identified by the first-pass approximation, the distance to the nearest parkrun would decrease by 6.6 km on average (SD = 54.2 km), and improve access to parkrun for 2.6 million people (10% of the population) living in 6,501 SA1s (see Table 2). The proportions of the population living within 1 km, 5 km and 10 km of an event are 5.3%, 67.9% and 88.1%, respectively; only 4.2% of the population live more than 25 km from an event. Improvements in access were greater for more deprived groups, as shown in Table 2; the least deprived group would have the average distance to the nearest parkrun reduced by 1.9 km, while the most deprived group would have distance reduced by 15.6 km.

Further investigation of Table 2 also reveals that very similar improvements in access can be achieved if locations are fine-tuned with either Method 1 (the SA1 that minimises IRSD-weighted distance from the SA2 centroid) or Method 2 (most densely populated SA1 within the SA2). Inspection of the interactive map shows that in many instances, the two methods select the same town. The full list of new sites selected, along with their catchment area population, can be found in Supplementary Table 2; we also list the motivations, methods and results of several sensitivity analyses which ultimately provided very similar recommendations for new sites as the methods presented here.

## Discussion

This study is the first analysis of geographic access to parkrun in Australia. We aimed to (1) assess the current access to public 5 km parkrun events across Australia, and stratified by socioeconomic disadvantage quintile, and (2) identify 100 new locations for parkrun events which would increase access for all Australians and in particular, those living in areas with the greatest socioeconomic disadvantage. Currently, 85.5% of the Australian population live within 10 km of an event, but there was a strong socioeconomic gradient, with the most disadvantaged living further away (an average 27.0 km to the nearest event). Starting 100 new events at the locations selected by our algorithm would increase the population living within 10 km to 88.1%, and would have the greatest benefit to the most disadvantaged communities, with a reduction, on average, to 11.4 km to the nearest event.

For this study, we closely followed the methods of Schneider et al. [12], who performed a similar study in the English context. Although we accessed and modified their open source code, several important differences in the English vs. Australian data and analysis approach are relevant here. The most obvious difference is the size of the geographic areas and populations under study; England covers 130,279 square km, has a population of over 66 million, and the longest distance to the nearest event was 76 km [12], while Australia covers more than 7.6 million square km, has a population of around 25 million [14], and the longest distance to an event was more than 2,200 km. That is, the Australian population density is considerably lower than that of England, and there are much larger tracts of relatively uninhabited land in the desert/outback. Schneider et al. [12] also used a database of public parks of more than 0.1 square km in size; no such database exists for Australia and hence we developed a two-step process to identify first a suitable region and then narrow to a town/locality within that region which was likely to have sufficient population and infrastructure to support an event. A further, minor difference is that we did not use population-weighted centroids as indicators of population location.

We also share some limitations with Schneider et al.’s study [12]. Distances are calculated “as the crow flies” and do not take into account natural or constructed boundaries such as lakes, mountains, or road access. It is particularly important to note that several new event locations are on islands; some of these are very remote, with access only by air, while others are less so, and can be accessed by air or ferry. New community physical activity events at those locations may initially be utilised only by people living on those islands; however, over time patronage may increase as participants may travel specifically to take part in these events.

Our two-step method was intended to provide the most detailed information possible without actually visiting an area. Limiting the selected locations to relatively densely populated SA1s provides a better chance, but does not guarantee, that the identified location will have suitable green space or critical mass of population required to support an event. Indeed, inspection of the interactive map for several very remote locations reveals very small communities sometimes without green space. Furthermore, even where green space is indicated, our map does not show information about whether the terrain is suitable for an event; Schneider et al. [12] raised similar concerns about their identified green spaces having possibly unsuitable terrain for an event. Lastly, the algorithm does not consider other factors which may also impact the success of starting a new event in that location, including the availability of people to lead the event (e.g., volunteering as route markers, etc.), attitudes towards and ability to exercise in that location (including very hot and/or humid environments), and the age of the population in the surrounding area. Choosing more specific locations will require local knowledge of amenities, environment, and the population. The success of starting new parkrun events at these locations will also depend on interaction with local community leaders. Reducing the barrier of distance will go some way to increasing physical activity levels among disadvantaged communities, but further strategies will be required to engage communities with parkrun.

While starting new parkrun events could help reduce the socioeconomic disparity in access and participation, a strategic mix of government policies is also needed. Progress needs a coordinated and strategic systems approach as outlined in the WHO Global Action Plan on Physical Activity 2018–2030 [5] and in the 2021 WHO advocacy brief Fair Play [6] which places particular emphasis on three areas of action (i) innovative and diverse financing mechanisms; (ii) coherent policy, laws, regulatory frameworks, and standards; and (iii) more integrated delivery of physical activity.

## Conclusions

This study provides strategic location suggestions for new parkrun locations in Australia that will improve equity of access to community physical activity events. In turn this could contribute to enabling greater participation in physical activity by disadvantaged communities reinforcing the critical role parkrun can play in reducing the inequalities in physical activity. A coordinated and strategic systems approach at a population level is required to increase physical activity in Australia and globally.

## Supplementary Information

**Additional file 1. **

**Additional file 2. **

**Additional file 3: Supplementary Table 1.** Predicted 2020 population, number of existing Australian 5 km parkrun events (at July 2021), and number of proposed parkrun events, in greater capital city and regional areas for each state. **Supplementary Figure 1a.** Map of current and proposed events for the greater capital city (Sydney) region of New South Wales. For this and all subsequent figures, numbered locations correspond to the order of selection by the location-allocation algorithm listed in Supplementary Table 2; the same population density scale has been used for all figures. **Supplementary Figure 1b.** Map of current and proposed events for regional New South Wales (NSW). Note for this and subsequent regional maps, event locations in the greater capital city are suppressed because of heavy clustering. Note also that the locations selected by the algorithm represent only the centroid of the SA2 area; sometimes this coincides with a regional town, but often the nearest town is visible as a darker purple area indicating higher population density. See the online interactive map for further details. **Supplementary Figure 2**. Map of current and proposed events for the Northern Territory (NT). **Supplementary Figure 3.** Map of current and proposed events for Queensland (QLD). **Supplementary Figure 4**. Map of current and proposed events for South Australia (SA). **Supplementary Figure 5.** Map of current and proposed events for Tasmania (TAS). **Supplementary Figure 6a.** Map of current and proposed events for the greater capital city (Melbourne) region of Victoria. **Supplementary Figure 6b.** Map of current and proposed events for regional Victoria (VIC). Supplementary Figure 7a. Map of current and proposed events for the greater capital city (Perth) region of Western Australia. Supplementary Figure 7b. Map of current and proposed events for regional Western Australia (WA). **Supplementary Figure 8.** Map of current events for the Australian Capital Territory (ACT). Note that no new events were proposed. **Supplementary Table 2.** Locations of new events selected by the location-allocation algorithm.

**Additional file 4.**

## Data Availability

With the exception of the location of current Australian parkrun events, all data are publicly available, and the R code to generate results is included as supplementary material. SA1 and SA2 ESRI shapefiles: https://www.abs.gov.au/AUSSTATS/abs@.nsf/DetailsPage/1270.0.55.001July%202016?OpenDocument#Data SA1 population data: Excel file downloadable from: https://www.data.qld.gov.au/dataset/erp-sa1-aus-consult SA1, SA2 and postcode level SEIFA data, and correspondence between SA1 and postcodes: https://www.abs.gov.au/AUSSTATS/abs@.nsf/DetailsPage/2033.0.55.0012016?OpenDocument

## Abbreviations

IQR: Interquartile range
IRSD: Index of Relative Socio-economic Disadvantage
SA1: Statistical area level 1
SA2: Statistical area level 2
SEIFA: Socio-economic Index for Area
