# Supplementary material for: Improving access to public physical activity events for disadvantaged communities in Australia
Source: BMC Public Health. 2022 Aug 13;22:1542. doi: 10.1186/s12889-022-13981-5 (PMC9375319; doi:10.1186/s12889-022-13981-5)
Supplement: Supplementary file 1 — Additional file 1. [file 12889_2022_13981_MOESM1_ESM.pdf]

Improving access to public physical activity events for disadvantaged communities in Australia: Supplementary Material

Smith, Reece, Rose & Owen

For our online map resource, white circles indicate the location of current parkrun events (at July 2021); red circles indicate the centroid of the SA2s selected in the first step of the analysis, together with the name of the SA2. Yellow circles indicate the location of the SA1 closest to the SA2 centroid, weighted by IRSD, while green circles indicate the most densely populated SA1 within the selected SA2 area. Numbers indicate the order in which the SA2 was selected, and all are also labelled by the name of the SA2.

Supplementary Table 1 shows the location of current events and proposed events by state and greater capital city/regional designation.

Supplementary Table 1. Predicted 2020 population, number of existing Australian 5 km parkrun events (at July 2021), and number of proposed parkrun events, in greater capital city and regional areas for each state.

	**Population**			**Number of current events**			**Number of new events**		
**State**	Capital city	Regional	Total	Capital city	Regional	Total	Capital city	Regional	Total
**New South Wales**	5,367,206	2,800,326	8,167,532	36	64	100	4	27	31
**Victoria**	5,159,211	1,537,459	6,696,670	42	50	92	3	5	8
**Queensland**	2,560,720	2,615,466	5,176,186	44	64	108	0	14	14
**Western Australia**	2,125,114	538,447	2,663,561	29	12	41	2	15	17
**South Australia**	1,376,601	393,774	1,770,375	19	16	35	0	10	10
**Tasmania**	238,834	301,946	540,780	5	12	17	0	3	3
**Australian Capital Territory**	431,380	0	431,380	7	-	7	0	0	0
**Northern Territory**	147,231	98,912	246,143	3	0	3	0	14	14
**Other territories**	0	4,671	4,671	-	0	0	-	3	3
**TOTAL**	17,406,297	8,291,001	25,697,298	185	218	403	9	91	100

Supplementary Figure 1a. Map of current and proposed events for the greater capital city (Sydney) region of New South Wales. For this and all subsequent figures, numbered locations correspond to the order of selection by the location-allocation algorithm listed in Supplementary Table 2; the same population density scale has been used for all figures.

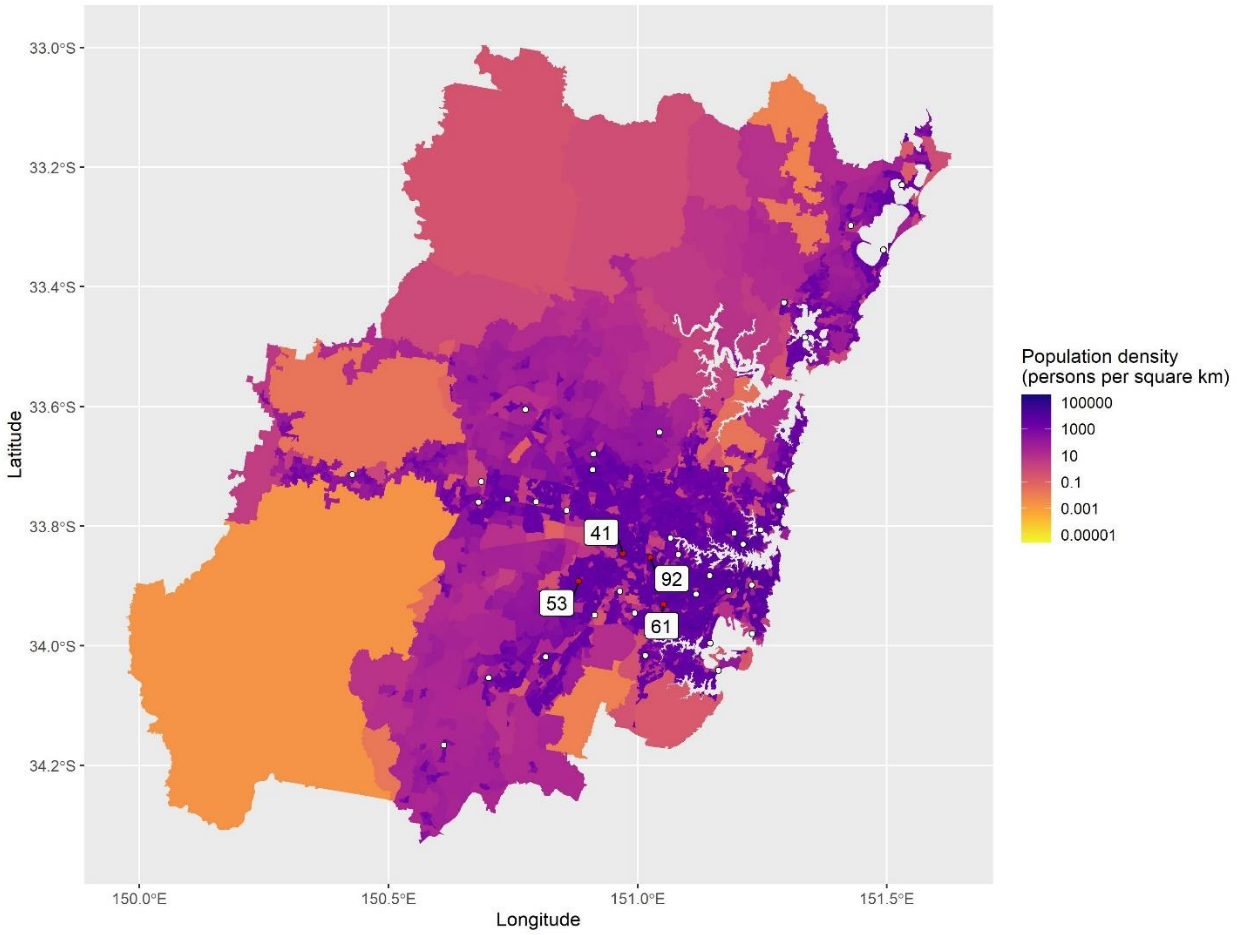

Supplementary Figure 1b. Map of current and proposed events for regional New South Wales (NSW). Note for this and subsequent regional maps, event locations in the greater capital city are suppressed because of heavy clustering. Note also that the locations selected by the algorithm represent only the centroid of the SA2 area; sometimes this coincides with a regional town, but often the nearest town is visible as a darker purple area indicating higher population density. See the online interactive map for further details.

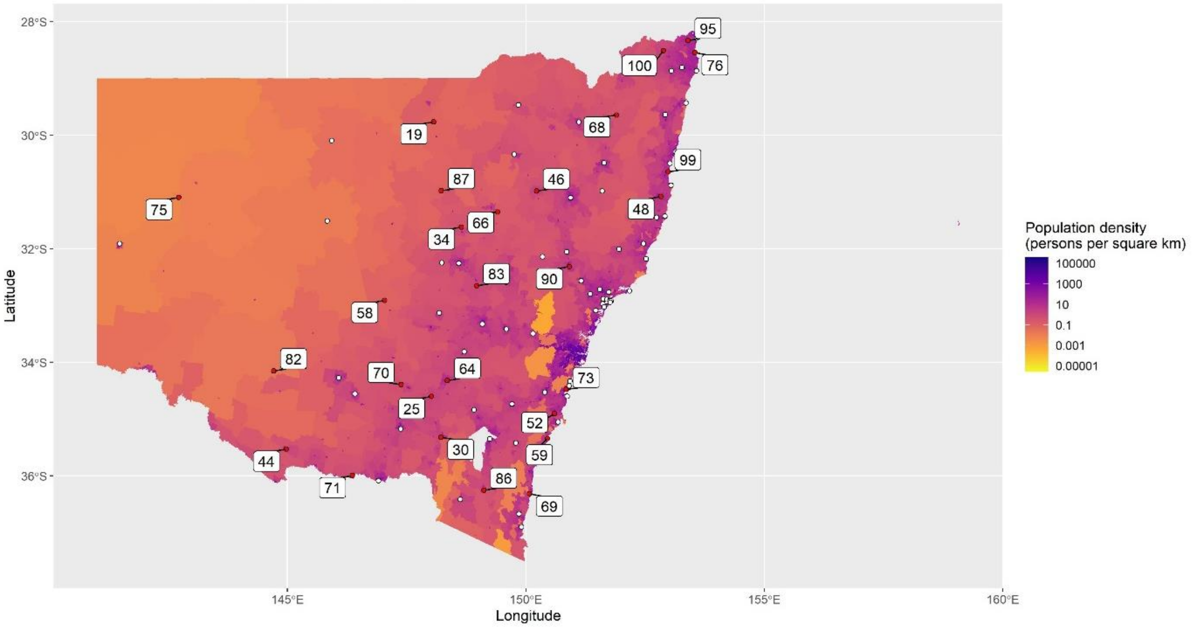

Supplementary Figure 2. Map of current and proposed events for the Northern Territory (NT).

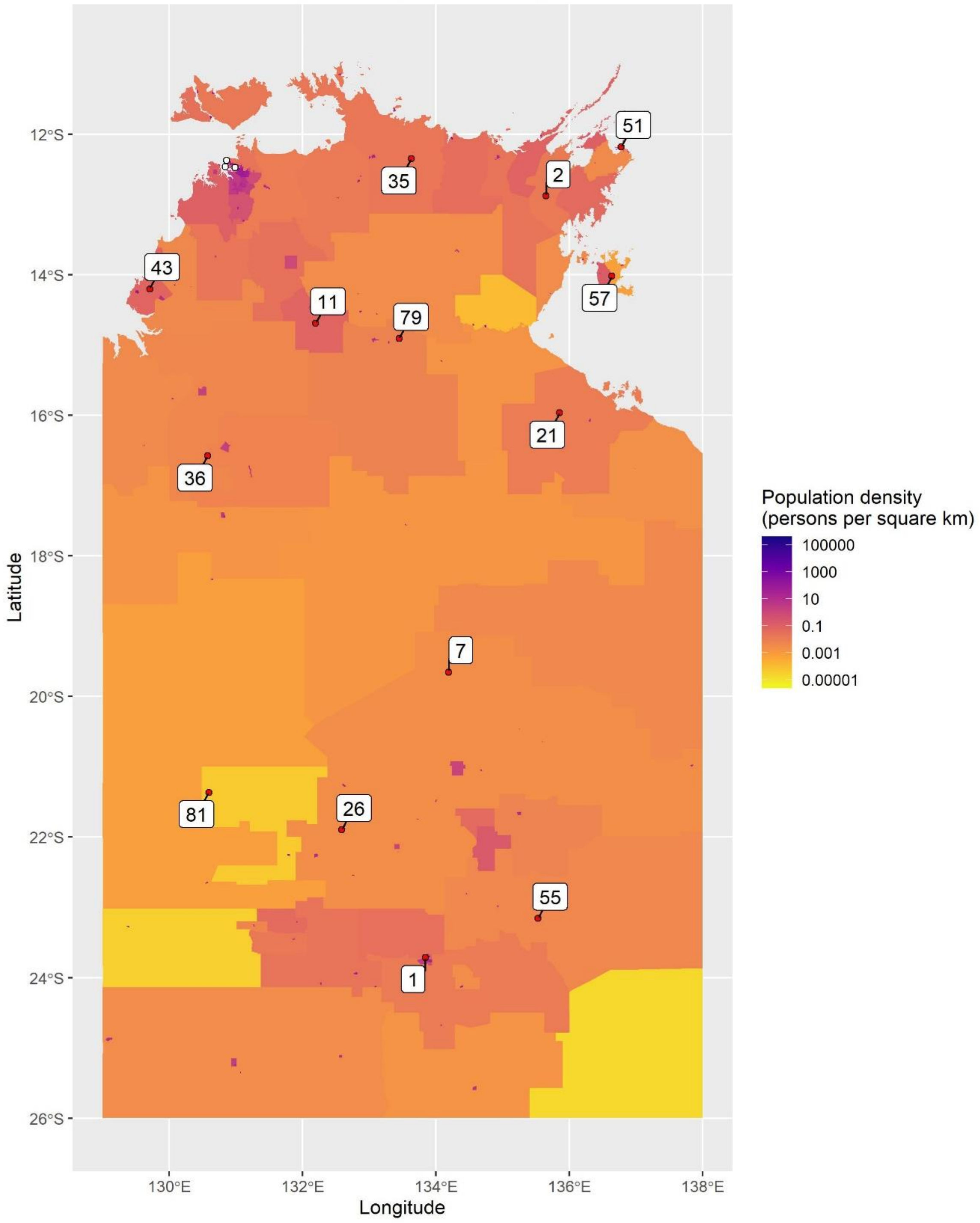

Supplementary Figure 3. Map of current and proposed events for Queensland (QLD).

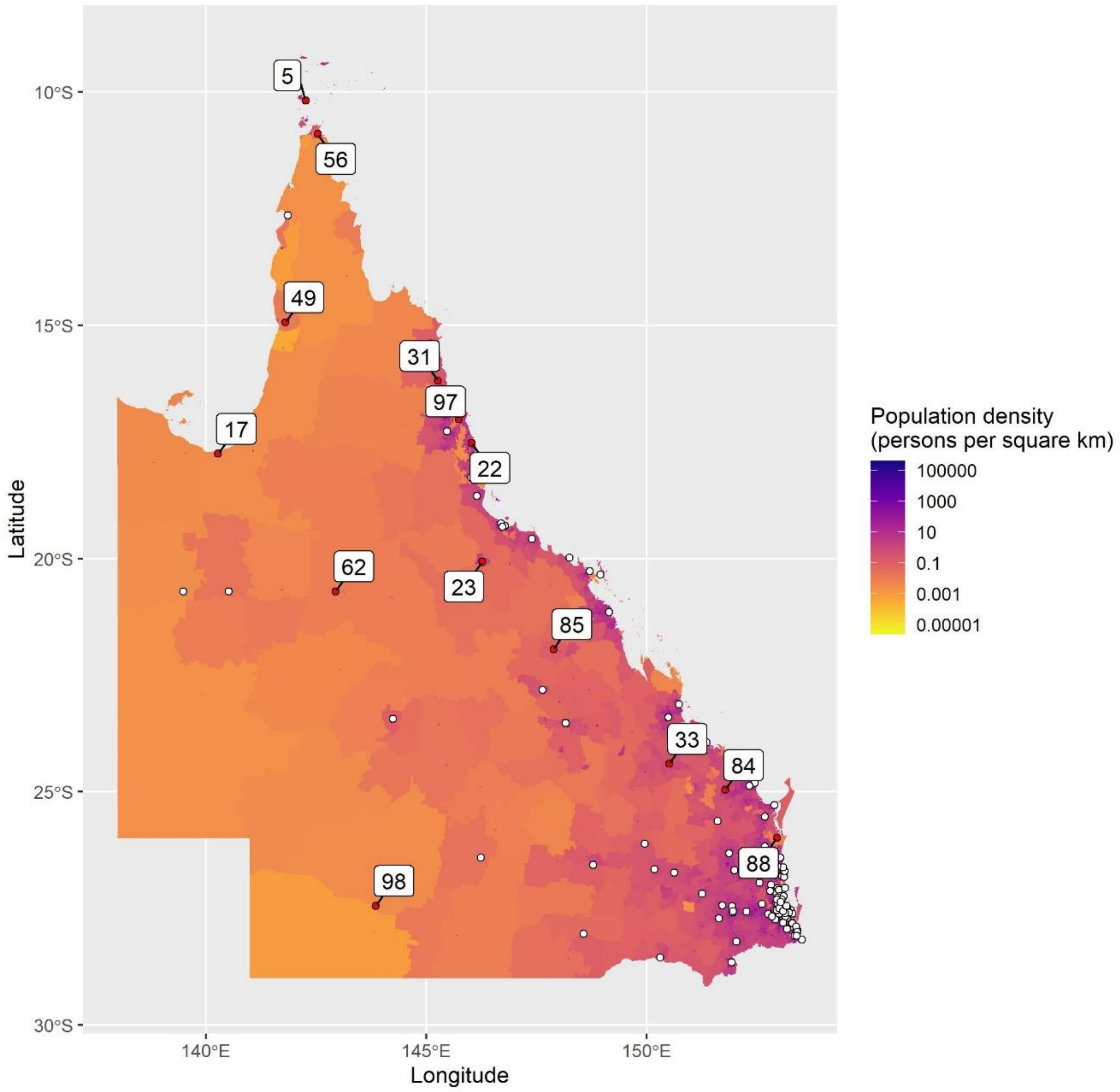

Supplementary Figure 4. Map of current and proposed events for South Australia (SA).

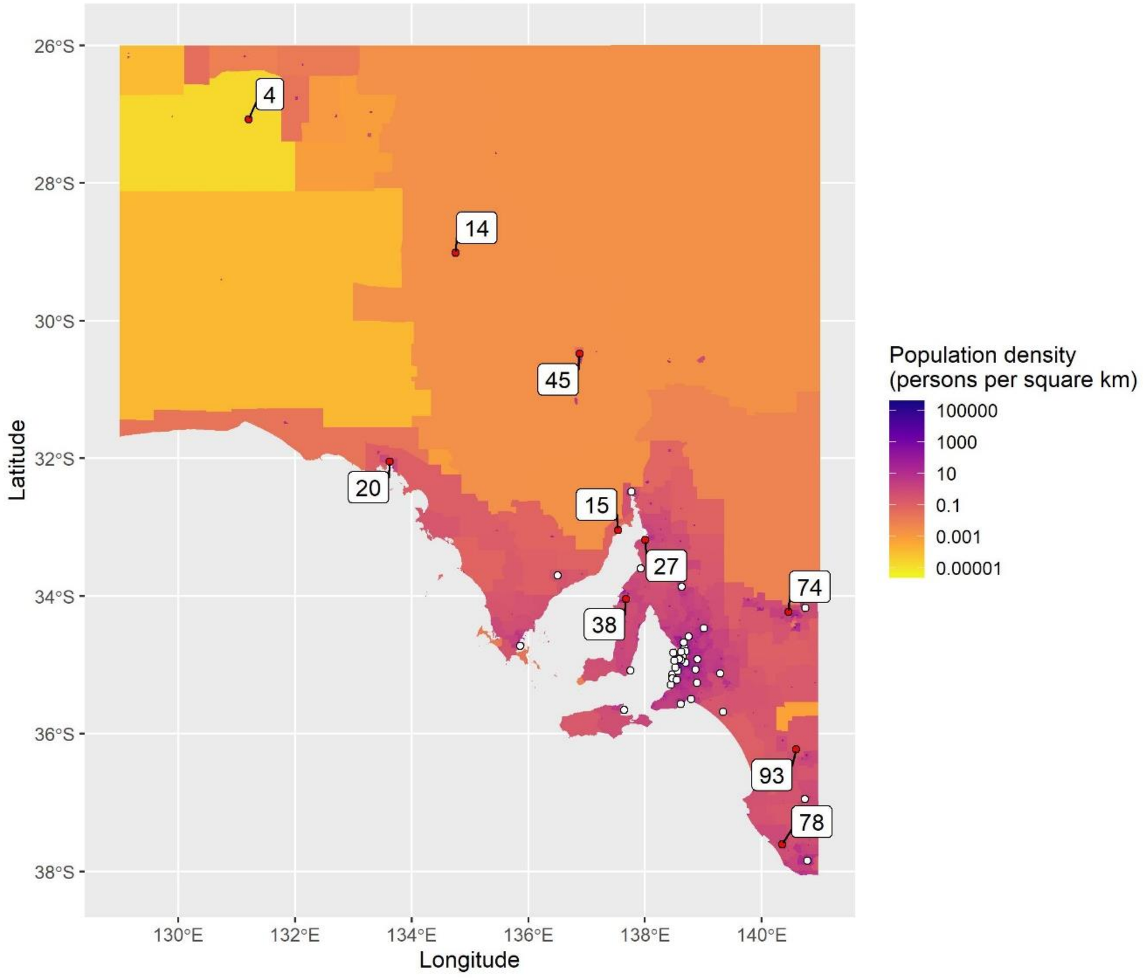

Supplementary Figure 5. Map of current and proposed events for Tasmania (TAS).

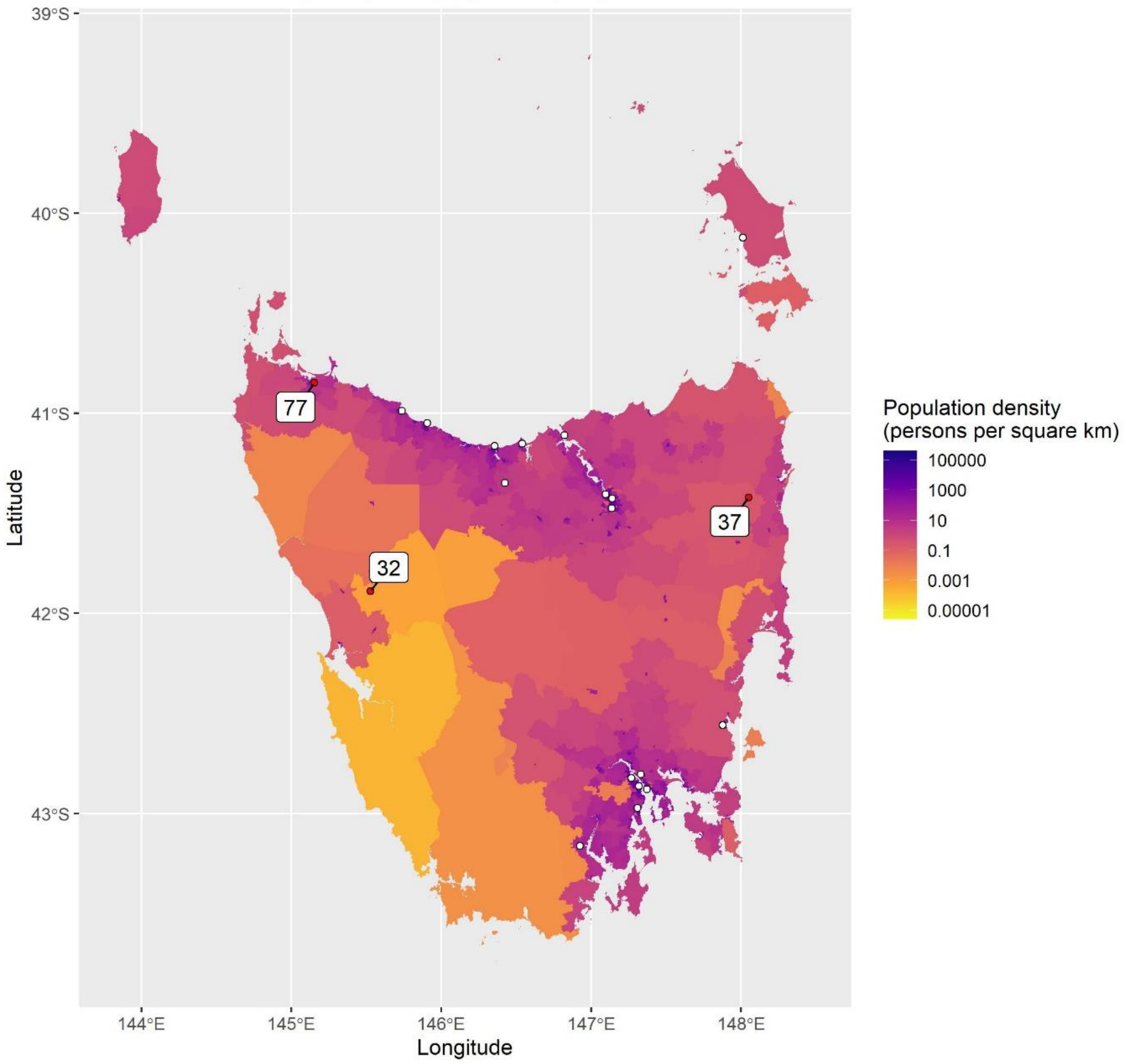

Supplementary Figure 6a. Map of current and proposed events for the greater capital city (Melbourne) region of Victoria.

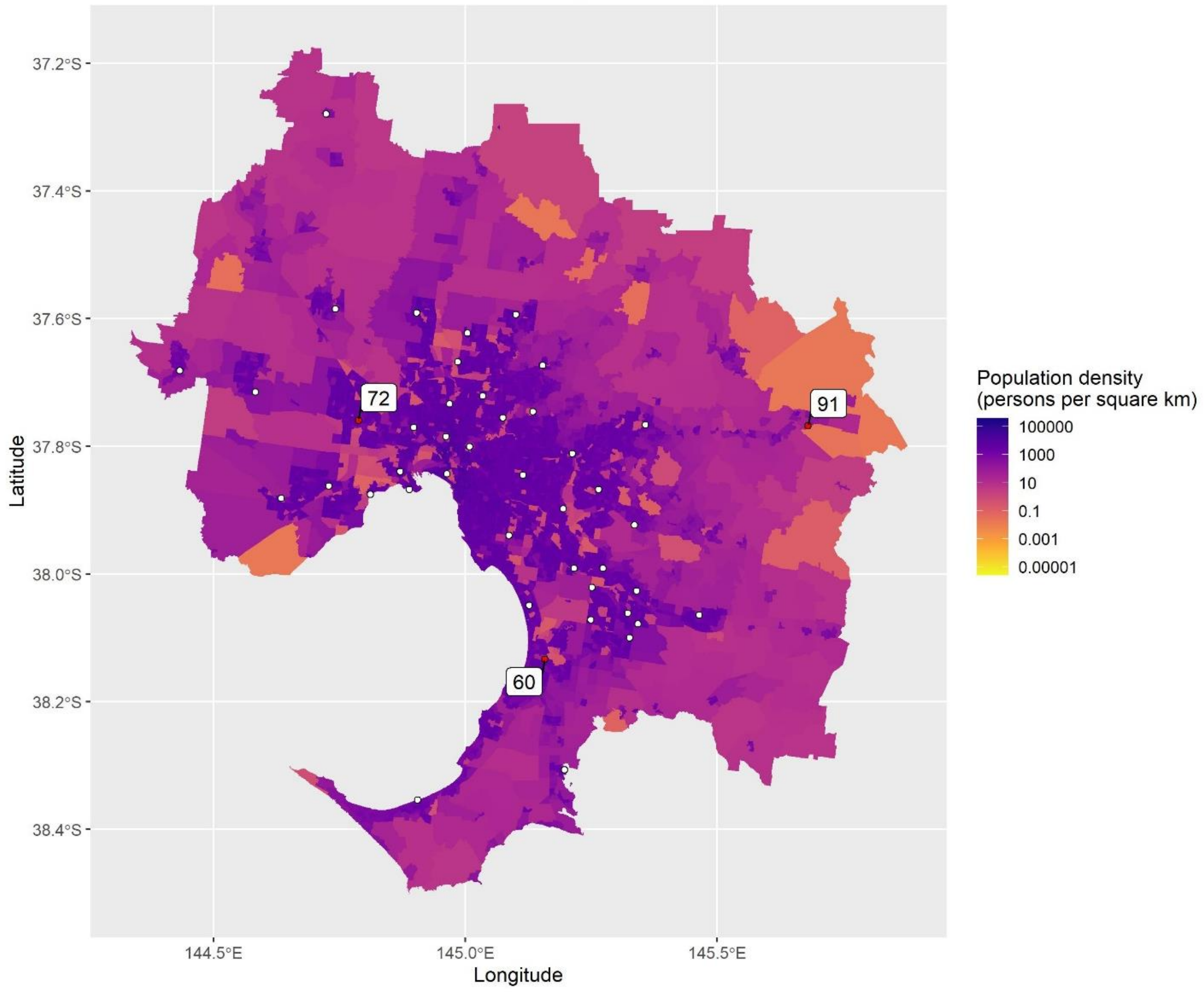

Supplementary Figure 6b. Map of current and proposed events for regional Victoria (VIC).

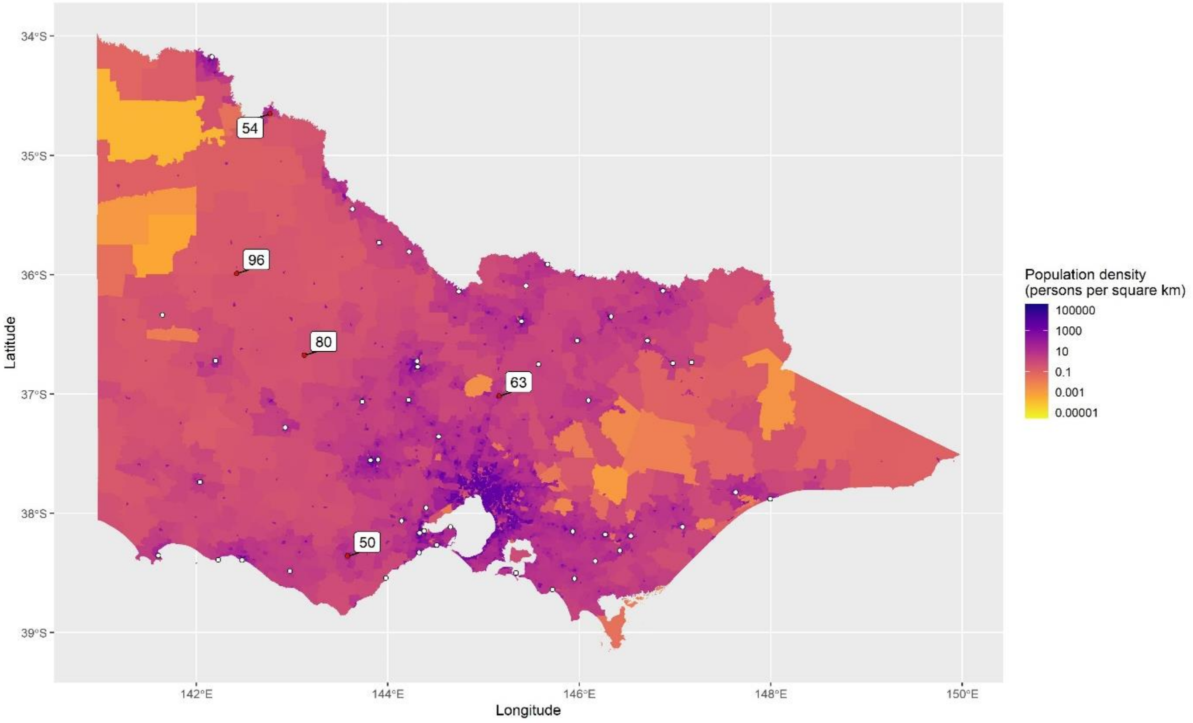

Supplementary Figure 7a. Map of current and proposed events for the greater capital city (Perth) region of Western Australia.

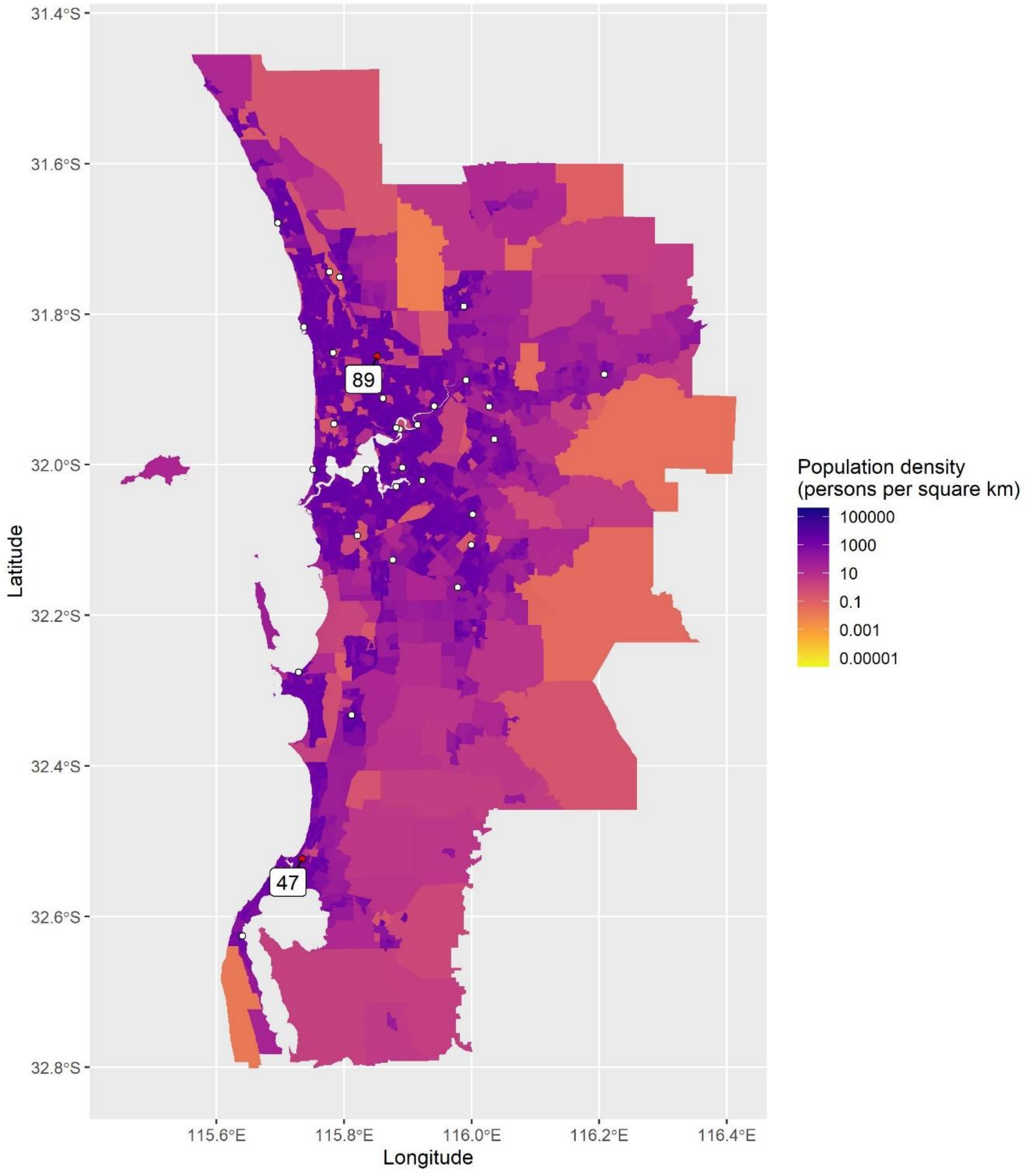

Supplementary Figure 7b. Map of current and proposed events for regional Western Australia (WA).

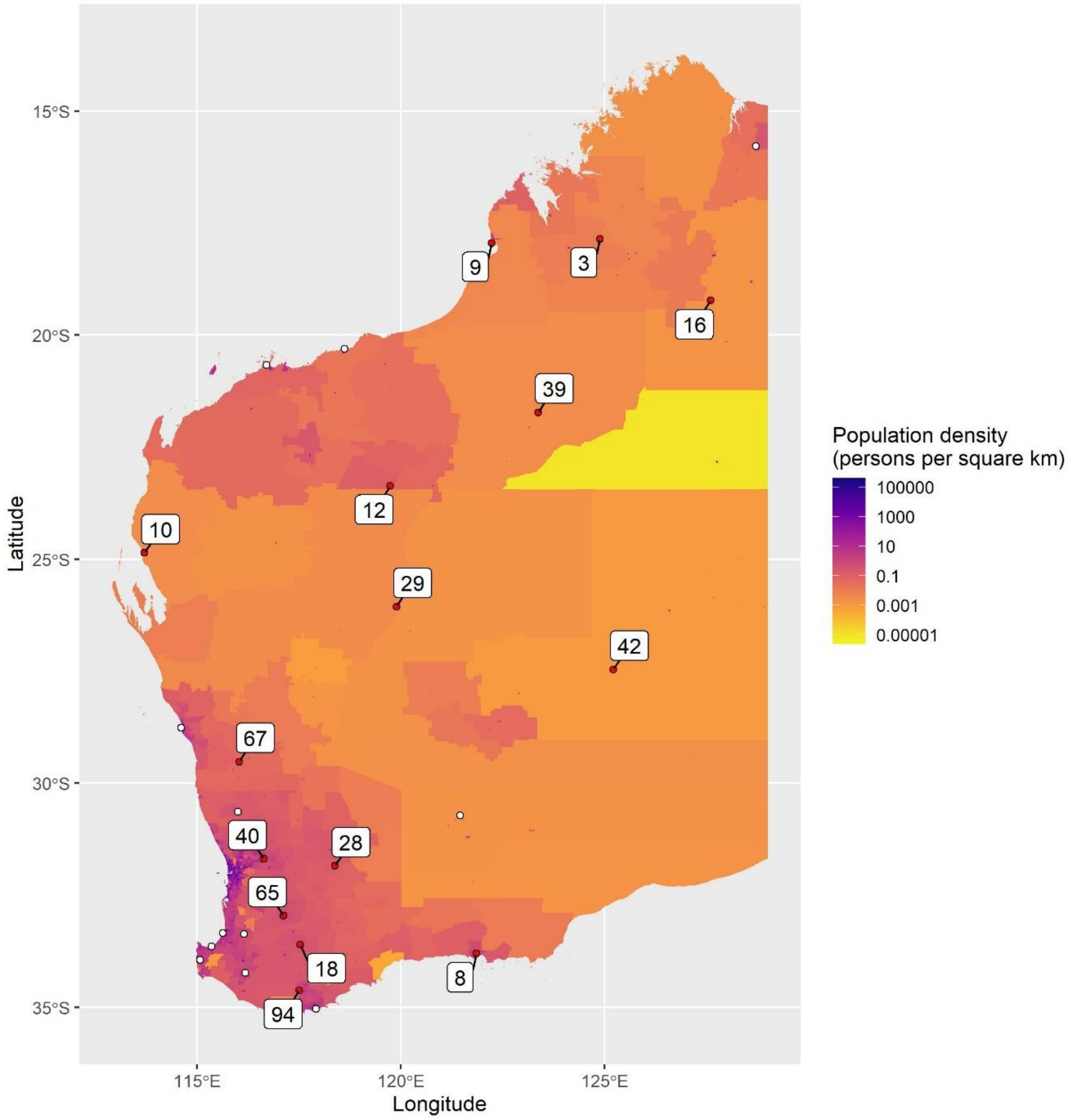

Supplementary Figure 8. Map of current events for the Australian Capital Territory (ACT). Note that no new events were proposed.

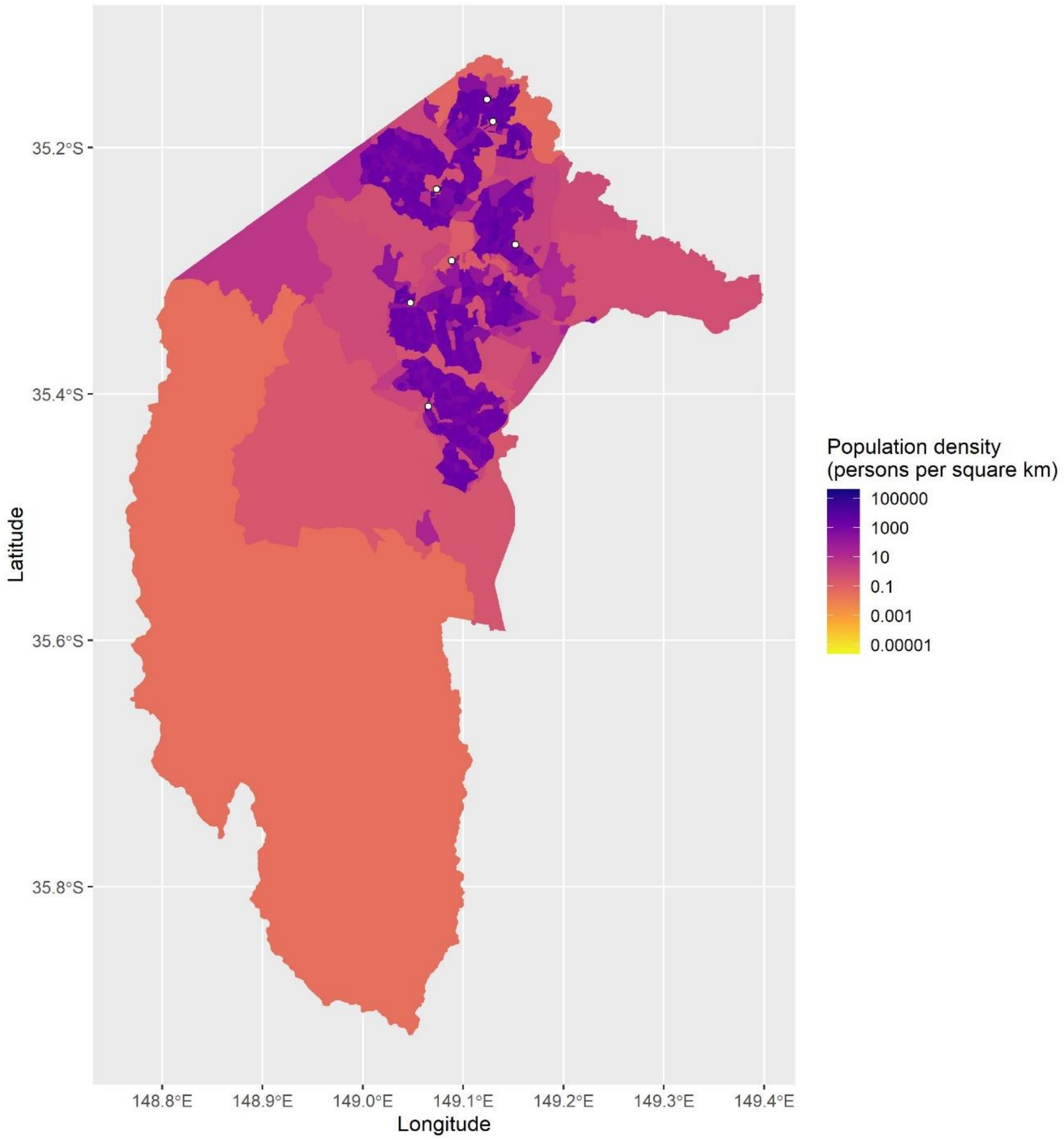

Supplementary Table 2. Locations of new events selected by the location-allocation algorithm.

Order	State	SA4	SA3	SA2	Town, Method 1*	Catchment population, Method 1*	Town, Method 2*	Catchment population, Method 2*
**1**	NT	Northern Territory - Outback	Alice Springs	Flynn (NT)	Larapinta Valley (Alice Springs)	30,642	Gillen (Alice Springs)	27,751
**2**	NT	Northern Territory - Outback	East Arnhem	East Arnhem	Gan Gan	6,981	Galiwinku	6,556
**3**	WA	Western Australia - Outback (North)	Kimberley	Derby - West Kimberley	Yungngora	4,680	Derby	7,270
**4**	SA	South Australia - Outback	Outback - North and East	APY Lands	Kaltjiti	4,280	Pukatja	6,188
**5**	QLD	Queensland - Outback	Far North	Torres Strait Islands	Yam Island	3,435	Coconut Island	3,195
**6**	OT	Other Territories	Christmas Island	Christmas Island	Flying Fish Cove	1,966	Flying Fish Cove	1,966
**7**	NT	Northern Territory - Outback	Barkly	Tennant Creek	Nyinkkanyunu (Tennant Creek)	5,570	Tennant Creek	5,570
**8**	WA	Western Australia - Outback (South)	Esperance	Esperance	Nulsen (Esperance)	15,956	Windabout (Esperance)	15,956
**9**	WA	Western Australia - Outback (North)	Kimberley	Broome	Broome	20,534	Djugan (Broome)	15,709
**10**	WA	Western Australia - Outback (South)	Gascoyne	Carnarvon	Morgantown (Carnarvon)	6,447	Brockman (Carnarvon)	6,447
**11**	NT	Northern Territory - Outback	Katherine	Katherine	Katherine	12,122	Katherine East	11,649
**12**	WA	Western Australia - Outback (North)	East Pilbara	Newman	Newman	12,920	Newman	12,589
**13**	OT	Other Territories	Norfolk Island	Norfolk Island	Norfolk Island	1,734	Norfolk Island	1,734
**14**	SA	South Australia - Outback	Outback - North and East	Coober Pedy	Coober Pedy	2,489	Coober Pedy	2,489
**15**	SA	South Australia - Outback	Eyre Peninsula and South West	Whyalla	Whyalla	21,918	Whyalla	21,918
**16**	WA	Western Australia - Outback (North)	Kimberley	Halls Creek	Billiluna	3,141	Halls Creek	5,772
**17**	QLD	Queensland - Outback	Outback - North	Carpentaria	Doomadgee	5,124	Normanton	5,641
**18**	WA	Western Australia - Wheat Belt	Albany	Katanning	Katanning	11,690	Katanning	12,134
**19**	NSW	Far West and Orana	Bourke - Cobar - Coonamble	Walgett - Lightning Ridge	Walgett	6,145	Lightning Ridge	5,674
**20**	SA	South Australia - Outback	Eyre Peninsula and South West	Ceduna	Ceduna	6,313	Ceduna	6,313
**21**	NT	Northern Territory - Outback	Katherine	Gulf	Borroloola	1,717	Borroloola	1,717
**22**	QLD	Cairns	Innisfail - Cassowary Coast	Innisfail	Innisfail	23,491	Innisfail	22,906
**23**	QLD	Townsville	Charters Towers - Ayr - Ingham	Charters Towers	Charters Towers	11,103	Charters Towers	10,871
**24**	OT	Other Territories	Cocos (Keeling) Islands	Cocos (Keeling) Islands	Bantam	573	Bantam	573
**25**	NSW	Riverina	Wagga Wagga	Cootamundra	Cootamundra	9,140	Cootamundra	9,140
**26**	NT	Northern Territory - Outback	Alice Springs	Yuendumu - Anmatjere	Willowra	1,424	Nturiya	2,665
**27**	SA	Barossa - Yorke - Mid North	Mid North	Port Pirie	Port Pirie	25,481	Port Pirie	25,481
**28**	WA	Western Australia - Wheat Belt	Wheat Belt - North	Merredin	Bruce Rock	13,559	Merredin	11,475

Order	State	SA4	SA3	SA2	Town, Method 1*	Catchment population, Method 1*	Town, Method 2*	Catchment population, Method 2*
**29**	WA	Western Australia - Outback (South)	Mid West	Meekatharra	Wiluna	2,697	Meekatharra	1,871
**30**	NSW	Riverina	Tumut - Tumbarumba	Tumut	Tumut	17,291	Tumut	17,291
**31**	QLD	Cairns	Port Douglas - Daintree	Daintree	Mossman Gorge Aboriginal Community	20,069	Mossman	20,069
**32**	TAS	West and North West	West Coast	West Coast (Tas.)	Zeehan	4,395	Queenstown	4,415
**33**	QLD	Central Queensland	Biloela	Biloela	Biloela	15,480	Biloela	15,480
**34**	NSW	Far West and Orana	Dubbo	Gilgandra	Gilgandra	4,390	Gilgandra	4,974
**35**	NT	Northern Territory - Outback	Daly - Tiwi - West Arnhem	West Arnhem	Oenpelli	6,936	Maningrida	6,667
**36**	NT	Northern Territory - Outback	Katherine	Victoria River	Kalkarindji	2,050	Lajamanu	2,158
**37**	TAS	Launceston and North East	North East	St Helens - Scamander	St Helens	9,436	St Helens	9,436
**38**	SA	Barossa - Yorke - Mid North	Yorke Peninsula	Moonta	Moonta	20,662	Moonta Bay	20,662
**39**	WA	Western Australia - Outback (North)	East Pilbara	East Pilbara	Punmu	959	Nullagine	3,089
**40**	WA	Western Australia - Wheat Belt	Wheat Belt - North	Northam	Northam	21,483	Northam	22,219
**41**	NSW	Sydney - Parramatta	Merrylands - Guildford	Guildford West - Merrylands West	Merrylands West	273,430	Merrylands West	307,090
**42**	WA	Western Australia - Outback (South)	Goldfields	Leinster - Leonora	Warburton	2,491	Leinster	3,700
**43**	NT	Northern Territory - Outback	Daly - Tiwi - West Arnhem	Thamarrurr	Wadeye	4,560	Wadeye	4,560
**44**	NSW	Murray	Upper Murray exc. Albury	Deniliquin	Deniliquin	10,533	Deniliquin	10,533
**45**	SA	South Australia - Outback	Outback - North and East	Roxby Downs	Roxby Downs	5,340	Roxby Downs	5,340
**46**	NSW	New England and North West	Tamworth - Gunnedah	Gunnedah	Gunnedah	15,536	Gunnedah	15,536
**47**	WA	Mandurah	Mandurah	Mandurah	Mandurah	93,472	Mandurah	93,622
**48**	NSW	Mid North Coast	Kempsey - Nambucca	Kempsey	Kempsey	22,396	Kempsey	22,396
**49**	QLD	Queensland - Outback	Far North	Kowanyama - Pormpuraaw	Pormpuraaw	2,328	Kowanyama	1,957
**50**	VIC	Warrnambool and South West	Colac - Corangamite	Colac	Colac	20,709	Colac	20,870
**51**	NT	Northern Territory - Outback	East Arnhem	Nhulunbuy	Nhulunbuy	4,869	Nhulunbuy	5,101
**52**	NSW	Southern Highlands and Shoalhaven	Shoalhaven	Nowra	Nowra	52,590	Worrigee (Nowra)	52,972
**53**	NSW	Sydney - South West	Fairfield	Bonnyrigg Heights - Bonnyrigg	Bonnyrigg	205,235	Bonnyrigg	201,598
**54**	VIC	North West	Murray River - Swan Hill	Robinvale	Robinvale	8,331	Robinvale	8,513
**55**	NT	Northern Territory - Outback	Alice Springs	Sandover - Plenty	Engawala	2,250	outside Alice Springs	3,086
**56**	QLD	Queensland - Outback	Far North	Torres	Thursday Island	8,891	Thursday Island	9,131
**57**	NT	Northern Territory - Outback	East Arnhem	Anindilyakwa	Angurugu	3,554	Alyangula	3,747
**58**	NSW	Central West	Lachlan Valley	Condobolin	Condobolin	6,889	Condobolin	6,889

Order	State	SA4	SA3	SA2	Town, Method 1*	Catchment population, Method 1*	Town, Method 2*	Catchment population, Method 2*
**59**	NSW	Southern Highlands and Shoalhaven	Shoalhaven	Ulladulla	Ulladulla	23,075	Ulladulla	22,795
**60**	VIC	Mornington Peninsula	Frankston	Frankston North	Frankston North	173,328	Frankston	176,999
**61**	NSW	Sydney - Inner South West	Canterbury	Punchbowl	Punchbowl	246,554	Punchbowl	240,957
**62**	QLD	Queensland - Outback	Outback - North	Northern Highlands	Richmond	2,610	Hughenden	2,557
**63**	VIC	Hume	Upper Goulburn Valley	Seymour	Seymour	23,220	Seymour	23,009
**64**	NSW	Capital Region	Young - Yass	Young	Young	19,377	Young	19,377
**65**	WA	Western Australia - Wheat Belt	Wheat Belt - South	Narrogin	Narrogin	13,081	Narrogin	14,325
**66**	NSW	Far West and Orana	Dubbo	Coonabarabran	Binnaway	8,635	Coonabarabran	6,271
**67**	WA	Western Australia - Outback (South)	Mid West	Morawa	Morowa	4,456	Three Springs	4,385
**68**	NSW	New England and North West	Inverell - Tenterfield	Glen Innes	Glen Innes	9,491	Glen Innes	9,491
**69**	NSW	Capital Region	South Coast	Narooma - Bermagui	Wallaga Lake Heights	14,031	Kianga	14,643
**70**	NSW	Riverina	Wagga Wagga	Temora	Temora	12,157	Temora	12,157
**71**	NSW	Murray	Upper Murray exc. Albury	Corowa	Corowa	19,374	Corowa	18,421
**72**	VIC	Melbourne - West	Brimbank	Cairnlea	Cairnlea	220,735	Cairnlea	213,507
**73**	NSW	Illawarra	Dapto - Port Kembla	Berkeley - Lake Heights - Cringila	Berkeley	87,052	Cringila	91,979
**74**	SA	South Australia - South East	Murray and Mallee	Barmera	Barmera	23,571	Marbera	23,571
**75**	NSW	Far West and Orana	Broken Hill and Far West	Far West	Wilcannia	1,700	Wilcannia	1,700
**76**	NSW	Richmond - Tweed	Richmond Valley - Coastal	Brunswick Heads - Ocean Shores	Brunswick Heads	36,366	South Golden Beach	34,399
**77**	TAS	West and North West	West Coast	Smithton	Smithton	9,138	Smithton	9,138
**78**	SA	South Australia - South East	Limestone Coast	Millicent	Millicent	11,132	Millicent	10,222
**79**	NT	Northern Territory - Outback	Katherine	Elsey	Jilkminggan	4,302	Wugularr	4,825
**80**	VIC	North West	Grampians	St Arnaud	St Arnaud	8,608	St Arnaud	8,608
**81**	NT	Northern Territory - Outback	Alice Springs	Tanami	Nyirripi	3,353	Papunya	4,059
**82**	NSW	Murray	Lower Murray	Hay	Hay	2,969	Hay	2,969
**83**	NSW	Far West and Orana	Dubbo	Wellington	Wellington	9,935	Wellington	10,399
**84**	QLD	Wide Bay	Burnett	Gin Gin	Gin Gin	12,464	Gin Gin	12,464
**85**	QLD	Mackay - Isaac - Whitsunday	Bowen Basin - North	Moranbah	Moranbah	14,012	Moranbah	14,012
**86**	NSW	Capital Region	Snowy Mountains	Cooma	Cooma	10,527	Cooma	10,260
**87**	NSW	Far West and Orana	Bourke - Cobar - Coonamble	Coonamble	Coonamble	5,367	Coonamble	4,853
**88**	QLD	Wide Bay	Gympie - Cooloola	Cooloola	Cooloola Cove	7,143	Tin Can Bay	7,143

Order	State	SA4	SA3	SA2	Town, Method 1*	Catchment population, Method 1*	Town, Method 2*	Catchment population, Method 2*
**89**	WA	Perth - North West	Stirling	Balga - Mirrabooka	Balga	136,919	Balga	145,879
**90**	NSW	Hunter Valley exc Newcastle	Upper Hunter	Muswellbrook	Muswellbrook	16,860	Muswellbrook	16,860
**91**	VIC	Melbourne - Outer East	Yarra Ranges	Yarra Valley	Millgrove	28,045	Millgrove	28,045
**92**	NSW	Sydney - Parramatta	Auburn	Auburn - Central	Auburn	161,148	Auburn	143,445
**93**	SA	South Australia - South East	Limestone Coast	Tatiara	Bordertown	9,106	Bordertown	9,106
**94**	WA	Western Australia - Wheat Belt	Albany	Plantagenet	Kendenup	7,214	Mount Barker	13,679
**95**	NSW	Richmond - Tweed	Tweed Valley	Murwillumbah	Murwillumbah	20,428	Murwillumbah	19,970
**96**	VIC	North West	Grampians	Yarriambiack	Beulah	7,874	Warracknabeal	7,679
**97**	QLD	Cairns	Cairns - South	Edmonton	Edmonton	51,815	Edmonton	56,264
**98**	QLD	Queensland - Outback	Outback - South	Far South West	Cunnamulla	1,874	Cunnamulla	1,874
**99**	NSW	Mid North Coast	Kempsey - Nambucca	Nambucca Heads	Nambucca Heads	18,685	Nambucca Heads	18,685
**100**	NSW	Richmond - Tweed	Richmond Valley - Hinterland	Kyogle	Kyogle	11,597	Kyogle	11,597

Order represents the order in which the algorithm selected the site; locations are given by state, the higher-level SA3 and SA4 areas, name of the SA2 area selected, towns selected by each method, and the catchment population for an event held at that location. Served population represents the number of people for whom this event would be the closest, after all 100 events had been set up, and in addition to the current 403 events. SA3s group whole SA2s with similar regional characteristics, while SA4s, made of whole SA3 areas, represent labour markets within each state or territory; for further detail see https://www.abs.gov.au/ausstats/abs@.nsf/Lookup/by%20Subject/1270.0.55.001~July%202016~Main%20Features~Main%20structure~10002

* Method 1: deprivation-weighted distance; Method 2: highest density.

To assess the sensitivity of the algorithm, we also ran analyses which only minimised distance to the nearest event (i.e., unweighted by IRSD) and found largely similar results to the analyses presented above: 156 of the 200 SA2 areas selected as locations for new events were the same in both analyses. The two analyses differed in the ordering of many locations, and many of the new locations selected in this analysis are close to areas selected above.
